# Trastuzumab Deruxtecan in Previously Treated HER2-Low Metastatic Breast Cancer: Real-World Multicentric Study in the Portuguese Population

**DOI:** 10.3390/cancers17121911

**Published:** 2025-06-09

**Authors:** Luísa Soares Miranda, Maria João Sousa, Miguel Martins Braga, Marisa Couto, Isabel Vieira Fernandes, Francisca Abreu, Inês Eiriz, Catarina Lopes Fernandes, Alice Fonseca Marques, Maria Teresa Marques, Raquel Romão, Fernando Gonçalves, Joana Simões, António Araújo

**Affiliations:** 1 Serviço de Oncologia Médica, Centro Hospitalar Universitário de Santo António, Unidade Local de Saúde de Santo António, 4099-001 Porto, Portugal; mjsousa@chporto.min-saude.pt (M.J.S.); miguelbraga.oncologia@chporto.min-saude.pt (M.M.B.); r.raquelromao@gmail.com (R.R.); fernando.goncalves@chporto.min-saude.pt (F.G.); joanasimoes.oncologia@chporto.min-saude.pt (J.S.); antonio.araujo@chporto.min-saude.pt (A.A.); 2 Oncology Research UMIB-Unit for Multidisciplinary Research in Biomedicine, ICBAS-School of Medicine and Biomedical Sciences, University of Porto, 4050-313 Porto, Portugal; 3 Laboratório para a Investigação Integrativa e Translacional em Saúde Populacional (ITR), University of Porto, 4050-600 Porto, Portugal; 4 Serviço de Oncologia Médica, Unidade Local de Saúdo de São João, 4200-319 Porto, Portugal; coutomarisa18@gmail.com; 5 Serviço de Oncologia Médica, Instituto Português de Oncologia de Coimbra Francisco Gentil, 3000-075 Coimbra, Portugal; 4148@ipocoimbra.min-saude.pt; 6 Serviço de Oncologia Médica, Hospital Senhora de Oliveira, Unidade Local de Saúde Alto Ave, 4835-044 Guimaraes, Portugal; franciscabreu@ulsaave.min-saude.pt; 7 Serviço de Oncologia Médica, Hospital Prof. Dr. Fernando Fonseca, Unidade Local de Saúde Amadora-Sintra, 2720-276 Amadora, Portugal; ines.eiriz@ulsasi.min-saude.pt; 8 Serviço de Oncologia Médica, Hospital Pedro Hispano, Unidade Local de Saúde de Matosinhos, 4464-513 Matosinhos, Portugal; catarina.lopesfernandes@ulsm.min-saude.pt; 9 Serviço de Oncologia Médica, Unidade Local de Saúde de Coimbra, 3004-561 Coimbra, Portugal; alicejfm@gmail.com; 10 Serviço de Oncologia Médica, Hospital de São Bernardo, Unidade Local de Saúde da Arrábida, 2910-446 Setúbal, Portugal; maria.t.marques@ulsa.min-saude.pt

**Keywords:** breast cancer, HER2-low, trastuzumab deruxtecan

## Abstract

**Background/Objectives**: Breast cancer is the most common malignant neoplasm in women and the leading cause of cancer-related death. Approximately 50% of HER2-negative breast cancers exhibit low expression of this protein (HER2-low). Trastuzumab deruxtecan (T-DXd) is an antibody-drug conjugate targeting the HER2 receptor which has shown benefit in patients with HER2-low metastatic breast cancer in the DESTINY-Breast04 study. However, few data are available on its efficacy in real-world practice. **Methods**: We conducted a retrospective multicenter national study (eight centers) including patients with advanced HER2-low breast cancer (immunohistochemistry 1+ or 2+/ in situ hybridization negative) who started T-DXd treatment between January 2022 and March 2024. Patients had received at least one previous line of treatment. The primary endpoint was real-world progression-free survival (rwPFS) in patients with metastatic HER2-low breast cancer treated with T-DXd. The secondary endpoints were real-world overall survival (OS) and objective response rate (ORR). **Results**: The study included 35 patients (34 female and 1 male patient), with a median age of 54 years at the start of T-DXd. All patients had an ECOG-PS 0–1, and 26 patients (74%) had hormone receptor (HR)-positive disease. The median number of prior lines of treatment was 4 [1–7], and 23 patients (65.8%) had metastases in three or more sites. With a median follow-up of 7.8 months, rwPFS was 6 months (95% CI, 2.3–9.7), and OS was 15 months (95% CI, 4.7–25.3). In HR-positive patients, the median rwPFS was 6 months (95% CI, 1.2–10.7), compared to 4 months (95% CI, 2.1–5.9) in HR-negative patients. The overall ORR was 52.9%. Adverse events of grade 3 or higher were neutropenia (2.9%) and fatigue (2.9%). **Conclusions**: This study provides real-world data on T-DXd in the treatment of advanced HER2-low breast cancer. It is noteworthy that the population was heavily pre-treated and had a higher proportion of HR-negative patients, which may explain the lower efficacy compared to the DESTINY-Breast04 study.

## Simple Summary

As antibody-drug conjugates continue to emerge as promising tools in cancer treatment, real-world evidence has become increasingly important in bridging the gap between results observed in controlled clinical trials and outcomes seen in everyday clinical practice. Such evidence helps to address ongoing uncertainties surrounding the adoption of new anticancer therapies in clinical settings. In this multicenter national study involving 35 patients, we aimed to evaluate the real-world efficacy of trastuzumab deruxtecan in HER2-low metastatic breast cancer, an area where limited real-world data currently exist.

## 1. Introduction

Breast cancer is the most diagnosed solid cancer and the leading cause of cancer-related deaths among women worldwide [1]. While breast cancer presents with diverse characteristics, advancements in understanding its molecular biology over the past thirty years have led to the development of targeted therapies, significantly improving patient outcomes [2].

Human epidermal growth factor receptor 2 (HER2) serves as a critical predictive and prognostic marker, often identified in both primary breast cancer tumors and distant metastases [3]. Historically, HER2 status was simplistically categorized into two groups: positive (score 3+ on immunohistochemical [IHC] analysis or IHC score 2+ with positive results on in situ hybridization [ISH]+) or negative (IHC 0, IHC 1+, or IHC 2+ ISH-) [4,5].

In the past, HER2-positive breast cancer was associated with an aggressive nature, frequent recurrences, and unfavorable survival rates. However, targeted therapies directed at the HER2 pathway have significantly improved outcomes. Trastuzumab was the pioneering medication developed to target the HER2 pathway [6,7]. More recently, attention has turned to a distinct subset of tumors within the HER2-negative category—referred to as HER2 low—comprising nearly 60% of metastatic breast cancers [8]. These tumors, which include both hormone receptor-positive and hormone receptor-negative subtypes, demonstrate heterogeneity in prognosis and in their response to systemic treatments [8]. Until recently, patients with HER2-low metastatic breast cancer had no HER2 target therapies available. The approval of trastuzumab deruxtecan, in August 2022, based on the DESTINY-Breast04 trial, marked the first targeted treatment option for these patients [9].

Trastuzumab deruxtecan (T-DXd) is an antibody-drug conjugate comprising an anti-HER2 monoclonal antibody, a cleavable tetrapeptide-based linker, and a potent cytotoxic topoisomerase inhibitor as payload [10,11]. In addition to its impressive results in HER2-positive populations [12], data from the DESTINY-Breast04 phase 3 clinical trial have also shown promising results for T-DXd in HER2-low metastatic breast cancer (mBC), defined as a score of 1+ on IHC or an IHC score of 2+ with negative results on ISH [13].

As clinical trials often have a more homogeneous patient population, real-world data includes a broader and more diverse patient population reflecting various demographics, comorbidities, and treatment histories. This diversity enhances our understanding of how the treatment performs in a broader patient context [14]. Therefore, our study was designed to investigate the real-world effectiveness, potential side effects, and prognostic determinants of T-DXd.

## 2. Material and Methods

This was a national, retrospective, multicenter study conducted in eight Portuguese medical oncology departments. This study aimed to characterize the real-world effectiveness and safety of T-DXd in patients with HER2-low mBC. Data were collected for patients who started T-DXd treatment between January 2022 and March 2024. The eight Portuguese medical oncology departments that participated were the following: Unidade Local de Saúde de Santo António, Unidade Local de Saúde de São João, Instituto Português de Oncologia de Coimbra Francisco Gentil, Unidade Local de Saúde Alto Ave, Unidade Local de Saúde Amadora-Sintra, -Unidade Local de Saúde de Matosinhos, Unidade Local de Saúde de Coimbra, and Unidade Local de Saúde da Arrábida. All hospitals had approval from their local ethics committees.

### 2.1. Data Collection

Patient data were collected from electronic medical records using a standardized data collection form developed by the coordinating center: Unidade Local de Saúde de Santo António. The collected variables included demographic data (age, gender, and race), clinical characteristics (Eastern Cooperative Oncology Group [ECOG] performance status, sites of metastasis, and number), pathological parameters (histological subtype, HER2 status, and hormonal receptor status), prior treatments (number and type of previous lines of therapy), and treatment-specific variables (T-DXd start and stop dates, dosage modifications, and adverse events graded according to CTCAE 5.0 criteria). Follow-up information comprised routine clinical evaluations and survival outcomes. All data were anonymized at the source, and each center provided only de-identified datasets, which were centrally compiled and reviewed by the coordinating center. Data quality was ensured through central consistency checks and clarification queries directed to participating centers when necessary.

### 2.2. HER2 Status Definition

HER2 expression was determined locally, at each site, based on standard institutional protocols. HER2-low status was defined as immunohistochemistry (IHC) score 1+ or 2+ with negative in situ hybridization (ISH) testing, in accordance with DESTINY-Breast04 criteria.

### 2.3. Patient Population

The eligibility criteria were as follows: age > 18 years old, unresectable, or metastatic pathologically documented breast cancer, HER2-low, hormone receptor (HR)-positive or HR-negative, must have received at least one prior line of chemotherapy for metastatic disease or experienced disease recurrence during or within six months of completing adjuvant chemotherapy. Patients with HR–positive disease must have received at least one line of endocrine therapy. Exclusion criteria were patients with incomplete or inadequate medical records, patients with insufficient follow-up information, and prior pathology tests indicating HER2 positivity.

### 2.4. Study Aims and Endpoints

The primary objective of the study was to evaluate the efficacy of T-DXd in a real-world population of patients with HER2-low mBC. Considering the real-world data collected in our study, we selected real-world PFS (rwPFS) as the primary endpoint. RwPFS was defined as the time from start of treatment to evidence of disease progression or death, whichever occurred first, in a clinical practice context, with no strict protocols for radiological evaluations and centralized reviews of images. Patients without an rwPFS event were censored at the last time they were known to be alive and free from progression.

Secondary endpoints were overall survival (OS) and objective response rate (ORR). OS was defined as the time from the start of T-DXd treatment to death; patients without an OS event were censored at the last time they were known to be alive. ORR was defined as complete plus partial responses. Furthermore, adverse events (AEs) and safety profile were evaluated. Treatment-related AEs collected in the referral centers were categorized and graded according to the National Cancer Institute Common Terminology Criteria for Adverse Events (NCI CTCAE), version 5.0.

### 2.5. Statistical Analyses

All data were extracted from the patients’ medical records and were analysed in a specifically designed database of the SPSS statistical package (SPSS Inc., Armonk, NY, USA) version 28. Baseline demographic and clinicopathological characteristics were recorded at time of enrolment, including age at initial diagnosis, type of malignancy, histology, stage at diagnosis, grade, HER2 status, ECOG performance status (PS), and prior treatments and their duration. Mean and standard deviation (SD) as well as median (interquartile range [IQR]) were used to describe continuous variables.

Tumor response was evaluated by the Response Evaluation Criteria in Solid Tumors (RECIST) version 1.1. The PFS and OS distributions were estimated using the Kaplan–Meier method and differences in survival were evaluated by the log-rank test. Point estimates of median survival and the associated Brookmeyer and Crowley 95% confidence interval (CI) estimates are reported when appropriate.

## 3. Results

Patient characteristics are reported in Table 1. A total of 35 patients were included in this study, 34 female and 1 male, with a median age of 54 years old (40–76) at the beginning of T-DXd treatment. All patients had an ECOG PS of 0–1, with 26 (74%) being HR-positive and 9 (26%) HR-negative. T-DXd was administered as third (14.3%), fourth (40%), and as fifth or later line (31.5%) in 85.8% of patients and the median number of prior treatment lines was four (one–seven). In total, 23 patients (65.8%) had metastases in three or more locations.

### 3.1. Efficacy

**Figure 1:**
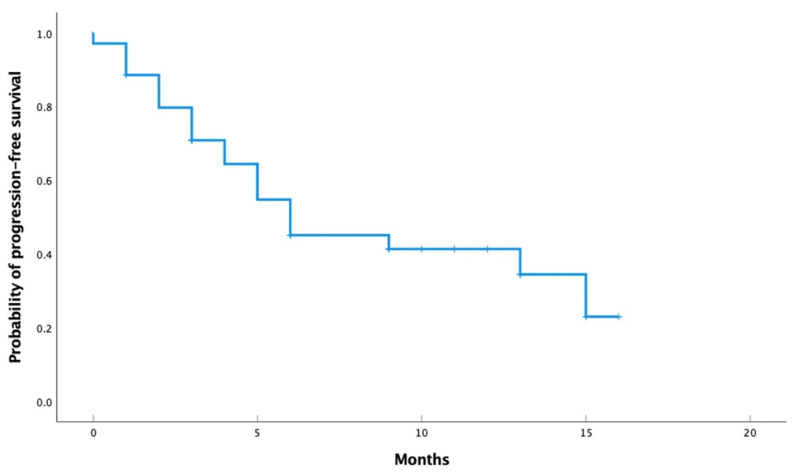
Real-world progression-free survival among all patients.

**Figure 2:**
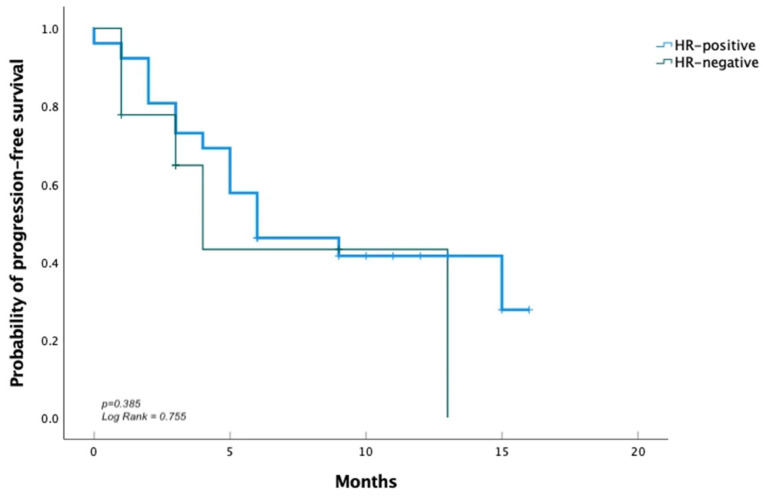
Real-world progression-free survival among HR-positive and HR-negative patients.

After a median follow-up period of 7.8 months, among all patients, median rwPFS was 6.0 months (95% CI, 2.3–9.7), and OS was 15.0 months (95% CI, 4.7–25.3) (Figure 1). The overall ORR was 52.9%. The median rwPFS was 6.0 months (95% CI, 1.2–10.7) in HR-positive patients, compared to 4.0 months (95% CI, 2.1–5.9) in HR-negative patients. (Figure 2).

### 3.2. Safety

During the follow-up, 80% of patients had at least one adverse event. The percentage of serious adverse events was 5.7% at grade 3 or higher (Table 2). The incidence of adverse events associated with discontinuation of treatment was 5.7%, and the incidence of adverse events associated with dose reductions and treatment delay was 14.3% and 17.1, respectively.

The most common adverse events of any grade included fatigue (57.0%), anorexia (37.1%), alopecia (31.4%) anemia, nausea (34.3%), and increased aminotransferase levels (25.7%). The most common adverse events of grade 3 or higher were neutropenia (2.9%) and fatigue (2.9%). Drug-related interstitial lung disease or pneumonitis were not observed in this population and neither were grade 5 events. Left ventricular dysfunction was reported in two patients (5.7%).

## 4. Discussion

T-DXd has recently emerged as a promising treatment option for HER2-low mBC, demonstrating impressive efficacy in the DESTINY-Breast04 trial. In this study, we observed lower rates of good outcomes among patients who had undergone extensive prior treatments; however, we found a similar ORR. The median number of previous treatment lines was four in our study and three in the DESTINY-Breast04 trial. Moreover, the proportion of HR-negative patients was 25.7% and the rate of brain metastasis was 17.1% whereas the numbers were 11.3% and 5.7%, respectively, in the DESTINY-Breast04 trial. As expected for a real-world cohort, these data indicate that the patients included in this study had distinct clinical characteristics compared to the patients in the DESTINY-Breast04 trial. This is possibly the main reason for the numerically lower median PFS noted in our study. Among all patients in the DESTINY-Breast04 trial, the median progression-free survival time was 9.9 months in the T-DXd group, while we observed 6.0 months in this study. In the DESTINY-Breast04 trial, in the HR-positive cohort the median progression-free survival time was 10.1 months and in the HR-negative patients it was 8.5 months. In our study, the median rwPFS in the HR-positive patients was 6.0 months, whereas in the HR-negative patients it was 4.0 months. Concerning OS, the DESTINY-Breast04 trial reported an OS of 23.4 months, and we observed an OS of 15.0 months. To the best of our knowledge, only a handful of studies have tested the real-life PFS and OS of T-DXd in HER2-low metastatic breast cancer. Our results are in line with those recent studies. One study presented at ASCO 2024 included HER2+, HER2-low, and HER2-0 metastatic breast cancer patients under T-DXd treatment [15]. Among HER2-low HR-positive patients, median PFS was 7.3 months (95% CI, 5.8–8.3), whereas in HR-negative patients it was 4.5 months (95% CI, 2.1–5.9). The OS among HER2-low patients was 15.5 months for HR-positive patients and 9.4 months for HR-negative patients. Additionally, our results are in line with DAISY [16], a phase II trial that studied T-DXd in a cohort of 73 HER2-low patients who received a median of five prior lines of therapy and had median PFS of 6.8 months and median OS of 11.6 months. In our study T-DXd showed favorable real-world activity for treating HER2-low metastatic breast cancer, although real-world PFS and OS seemed shorter than was observed in the DESTINY-Breast04 trial.

Historically, breast cancer prognosis and treatment were determined by a binary classification of HER2 status (positive or negative), primarily based on trastuzumab’s effectiveness in HER2-positive cases. The approval of T-DXd for HER2-low advanced breast cancer has transformed the treatment landscape, offering a targeted therapy option for more than half of mBC patients who previously lacked effective HER2-directed treatments. However, the HER2-low population encompasses both hormone receptor-positive and hormone receptor-negative subgroups, and studies in these groups are needed to provide valuable comparisons for understanding the characteristics of treatment responses and efficacy.

The DESTINY-Breast04 trial created a paradigm shift in mBC treatment by launching T-DXd as the first effective therapy for HER2-low mBC, particularly in hormone-resistant patients. Moreover, DESTINY-Breast06 [17] further expanded this paradigm by demonstrating that even patients with ultra-low HER2 expression (IHC 1+ or 0) can benefit from T-DXd, especially those who are endocrine-resistant, reinforcing its role as a post-endocrine but pre-chemotherapy option [17]. These findings challenge the traditional HER2 classification and expand the applicability of HER2-directed therapy beyond HER2-positive disease and opening a new possibility, namely for the treatment of hormone-resistant and chemo-naïve patients. However, in triple-negative breast cancer the role of T-DXd remains more limited. Based on the ASCENT trial [18] results, Sacituzumab govitecan is the preferred agent in second-line settings, with T-DXd being considered only from the third line forward and only for HER2-low patients. Current guidelines from ESMO [19], NCCN [20], and ASCO [21] reflect these developments by recommending T-DXd for hormone-positive mBC and triple-negative mBC in different settings. These developments highlight an important evolution in the management of mBC by introducing new therapeutic options for hormone-resistant and triple-negative mBC.

The safety profile of T-DXd in this study was manageable and no new safety concerns were observed. The most common adverse events of grade 3 or higher were neutropenia and fatigue. Left ventricular dysfunction was reported in two patients. In contrast with DESTINY-Breast04, no cases of drug-related interstitial lung disease or pneumonitis were observed. The absence of these adverse events in this study, compared to the trial, may be due to incomplete documentation in clinical registries or reflect a genuinely reduced occurrence of these events, and we cannot exclude either possibility.

The main strengths of our study are in addressing the current lack of real-world data on the efficacy of T- DXd in HER2-low metastatic breast cancer patients and the multicentric design. The major limitation is the limited sample size.

### Future Research Directions

Antibody-drug conjugates (ADCs) are emerging as a transformative approach in oncology by offering a unique method of delivering cytotoxic agents straight to cancer cells with high precision [22]. By coupling a monoclonal antibody specific to tumor-associated antigens with a potent cytotoxic payload, ADCs selectively deliver a potent cytotoxic agent to tumor cells, improving the therapeutic index of chemotherapeutic agents. Moreover, their bystander effect [23], where the released payloads also act on neighboring cells, intensify their therapeutic impact regardless of target cell expression [24,25]. Recent advancements have led to the approval of multiple ADCs for different cancers, including breast, lung, and ovarian, demonstrating improved efficacy and safety profiles compared to traditional chemotherapy [26]. The development of new linkers and payloads will improve the stability and specificity of ADCs, reducing off-target effects and improving therapeutic outcomes. As research progresses, ADCs are poised to redefine the landscape of chemotherapy, shifting from non-specific cytotoxicity to targeted, personalized treatment strategies that align with the principles of precision medicine. Transitioning ADCs into earlier treatment lines shows how standards of care are and will continue to be redefined, opening the possibility of combining ADCs with other existing treatments [27]. New ADCs are being developed with dual chemotherapy agents and immunotherapeutic activity, and these will certainly bring a new dimension to the field of ADCs.

## 5. Conclusions

In conclusion, this multicentric study reports real-world data of T-DXd in patients with heavily pre-treated HER2-low metastatic/unresectable breast cancer. T-DXd had antitumor activity with a similar response to that reported in previous clinical studies and was well tolerated. No new safety concerns were reported.

## Figures and Tables

**Table 1:** Demographic and clinical characteristics of patients at the beginning of T-DXd.

Characteristics	
Median age-yr (range)	54 (40–76)
Female sex-no (%)	34 (97.1)
Race-no (%)	
White	34 (97.1)
Black	1 (2.9)
HER2-low status-no (%) ^‡^	
IHC 1+	26 (74.3)
IHC 2+ and ISH-negative	9 (25.7)
ECOG performance status score-no (%) ^§^	
0	13 (37.1)
1	22 (62.9)
Hormone receptor-positive-no (%)	26 (74.3)
Metastasis-no (%)	
Brain	6 (17.1)
Bone	26 (74.3)
Visceral	29 (82.9)
Median no. of lines (range)	4 (1–7)
No of lines (%)	
1	1 (2.9)
2	4 (11.4)
≥3	30 (85.7)

^‡^ Low expression of human epidermal growth factor receptor 2 (HER2) was defined as a score of 1+ on immunohistochemical (IHC) analysis or as an IHC score of 2+ and negative results on in situ hybridization (ISH). ^§^ Performance status scores on the Eastern Cooperative Oncology Group (ECOG) scale range from 0 (no disability) to 5 (death).

**Table 2:** Most common drug-related adverse events.

Events	All Grade	Grade 1–2	Grade ≥3
Number of patients (percent %)			
**Blood and lymphatic system disorders**			
Neutropenia	5 (14.3%)	4 (11.4%)	1 (2.9%)
Anemia	12 (34.3%)	12 (34.3%)	0
Thrombocytopenia	3 (8.6%)	3 (8.6%)	0
**Gastrointestinal disorders**			
Nausea	12 (34.3%)	12 (34.3%)	0
Vomiting	7 (20.0%)	7 (20.0%)	0
Diarrhea	6 (17.1%)	6 (17.1%)	0
Constipation	4 (11.4%)	4 (11.4%)	0
**General disorders**			
Fatigue	22 (62.9%)	21(57.0%)	1 (2.9%)
Anorexia	13 (37.1%)	13 (37.1%)	0
**Skin and subcutaneous tissue disorders**			
Alopecia	11 (31.4%)	11(31.4%)	0
**Cardiac toxicity** (LVEF reduction)	2 (5.7%)	2 (5.7%)	0
**Hepatic toxicity** (increased aminotransferase levels)	9 (25.7%)	9 (25.7%)	0
**Pulmonary toxicity** (interstitial lung disease or pneumonitis)	0	0	0

## Data Availability

The data presented in this study are not publicly available due to ethical and legal restrictions related to patient confidentiality.

## References

[1] Filho A.M., Laversanne M., Ferlay J., Colombet M., Piñeros M., Znaor A., Parkin D.M., Soerjomataram I., Bray F. (2024). The GLOBOCAN 2022 cancer estimates: Data sources, methods, and a snapshot of the cancer burden worldwide. Int. J. Cancer.

[2] Xiong X., Zheng L., Ding Y., Chen Y., Cai Y., Wang L., Huang L., Liu C., Shao Z., Yu K. (2025). Breast cancer: Pathogenesis and treatments. Signal Transduct. Target. Ther..

[3] Slamon D.J., Clark G.M., Wong S.G., Levin W.J., Ullrich A., McGuire W.L. (1987). Human breast cancer: Correlation of relapse and survival with amplification of the HER-2/neu oncogene. Science.

[4] Ahn S., Woo J.W., Lee K., Park S.Y. (2020). HER2 status in breast cancer: Changes in guidelines and complicating factors for interpretation. J. Pathol. Transl. Med..

[5] Harbeck N., Penault-Llorca F., Cortes J., Gnant M., Houssami N., Poortmans P., Ruddy K., Tsang J., Cardoso F. (2019). Breast cancer. Nat. Rev. Dis. Primers.

[6] Swain S.M., Baselga J., Kim S.-B., Ro J., Semiglazov V., Campone M., Ciruelos E., Ferrero J.-M., Schneeweiss A., Heeson S. (2015). Pertuzumab, Trastuzumab, and Docetaxel in HER2-Positive Metastatic Breast Cancer. N. Engl. J. Med..

[7] Marty M., Cognetti F., Maraninchi D., Snyder R., Mauriac L., Tubiana-Hulin M., Chan S., Grimes D., Antón A., Lluch A. (2005). Randomized Phase II Trial of the Efficacy and Safety of Trastuzumab Combined With Docetaxel in Patients With Human Epidermal Growth Factor Receptor 2–Positive Metastatic Breast Cancer Administered As First-Line Treatment: The M77001 Study Group. J. Clin. Oncol..

[8] Tarantino P., Hamilton E., Tolaney S.M., Cortes J., Morganti S., Ferraro E., Marra A., Viale G., Trapani D., Cardoso F. (2020). HER2-Low Breast Cancer: Pathological and Clinical Landscape. J. Clin. Oncol..

[9] Roy A.M., Kumarasamy V.M., Dhakal A., O’regan R., Gandhi S. (2023). A review of treatment options in HER2-low breast cancer and proposed treatment sequencing algorithm. Cancer.

[10] Nakada T., Sugihara K., Jikoh T., Abe Y., Agatsuma T. (2019). The Latest Research and Development into the Antibody–Drug Conjugate, [fam-] Trastuzumab Deruxtecan (DS-8201a), for HER2 Cancer Therapy. Chem. Pharm. Bull..

[11] Ogitani Y., Aida T., Hagihara K., Yamaguchi J., Ishii C., Harada N., Soma M., Okamoto H., Oitate M., Arakawa S. (2016). DS-8201a, A Novel HER2-Targeting ADC with a Novel DNA Topoisomerase I Inhibitor, Demonstrates a Promising Antitumor Efficacy with Differentiation from T-DM1. Clin. Cancer Res..

[12] Bartsch R., Berghoff A.S., Furtner J., Marhold M., Bergen E.S., Roider-Schur S., Starzer A.M., Forstner H., Rottenmanner B., Dieckmann K. (2022). Trastuzumab deruxtecan in HER2-positive breast cancer with brain metastases: A single-arm, phase 2 trial. Nat. Med..

[13] Modi S., Jacot W., Yamashita T., Sohn J., Vidal M., Tokunaga E., Tsurutani J., Ueno N.T., Prat A., Chae Y.S. (2022). Trastuzumab Deruxtecan in Previously Treated HER2-Low Advanced Breast Cancer. N. Engl. J. Med..

[14] Sherman R.E., Anderson S.A., Dal Pan G.J., Gray G.W., Gross T., Hunter N.L., LaVange L., Marinac-Dabic D., Marks P.W., Robb M.A. (2016). Real-World Evidence—What Is It and What Can It Tell Us?. N. Engl. J. Med..

[15] Tarantino P., Lee D., Foldi J., Soulos P.R., Gross C.P., Grinda T., Winer E.P., Lin N.U., Krop I.E., Tolaney S.M. (2024). Outcomes with trastuzumab deruxtecan (T-DXd) by HER2 status and line of treatment in a large real-world database of patients with metastatic breast cancer. J. Clin. Oncol..

[16] Mosele F., Deluche E., Lusque A., Le Bescond L., Filleron T., Pradat Y., Ducoulombier A., Pistilli B., Bachelot T., Viret F. (2023). Trastuzumab deruxtecan in metastatic breast cancer with variable HER2 expression: The phase 2 DAISY trial. Nat. Med..

[17] Bardia A., Hu X., Dent R., Yonemori K., Barrios C.H., O’shaughnessy J.A., Wildiers H., Pierga J.-Y., Zhang Q., Saura C. (2024). Trastuzumab Deruxtecan after Endocrine Therapy in Metastatic Breast Cancer. N. Engl. J. Med..

[18] Bardia A., Hurvitz S.A., Tolaney S.M., Loirat D., Punie K., Oliveira M., Brufsky A., Sardesai S.D., Kalinsky K., Zelnak A.B. (2021). Sacituzumab Govitecan in Metastatic Triple-Negative Breast Cancer. N. Engl. J. Med..

[19] Gennari A., André F., Barrios C., Cortés J., de Azambuja E., DeMichele A., Dent R., Fenlon D., Gligorov J., Hurvitz S. (2021). ESMO Clinical Practice Guideline for the diagnosis, staging and treatment of patients with metastatic breast cancer. Ann. Oncol..

[20] Gradishar W.J., Moran M.S., Abraham J., Abramson V., Aft R., Agnese D., Allison K.H., Anderson B., Bailey J., Burstein H.J. (2024). Breast Cancer, Version 3.2024, NCCN Clinical Practice Guidelines in Oncology. J. Natl. Compr. Cancer Netw..

[21] Al Sukhun S., Koczwara B., Temin S., Arun B.K. (2024). Systemic Treatment of Patients With Metastatic Breast Cancer: ASCO Resource–Stratified Guideline Q and A. JCO Glob. Oncol..

[22] Liang Y., Zhang P., Li F., Lai H., Qi T., Wang Y. (2024). Advances in the study of marketed antibody-drug Conjugates (ADCs) for the treatment of breast cancer. Front. Pharmacol..

[23] Ogitani Y., Hagihara K., Oitate M., Naito H., Agatsuma T. (2016). Bystander killing effect of DS-8201a, a novel anti-human epidermal growth factor receptor 2 antibody–drug conjugate, in tumors with human epidermal growth factor receptor 2 heterogeneity. Cancer Sci..

[24] Géraud A., Gougis P., de Nonneville A., Beaufils M., Bertucci F., Billon E., Brisou G., Gravis G., Greillier L., Guerin M. (2025). Pharmacology and pharmacokinetics of antibody-drug conjugates, where do we stand?. Cancer Treat. Rev..

[25] Drago J.Z., Modi S., Chandarlapaty S. (2021). Unlocking the potential of antibody–drug conjugates for cancer therapy. Nat. Rev. Clin. Oncol..

[26] Tarantino P., Pestana R.C., Corti C., Modi S., Bardia A., Tolaney S.M., Cortes J., Soria J., Curigliano G. (2021). Antibody–drug conjugates: Smart chemotherapy delivery across tumor histologies. CA A Cancer J. Clin..

[27] Nicolò E., Giugliano F., Ascione L., Tarantino P., Corti C., Tolaney S.M., Cristofanilli M., Curigliano G. (2022). Combining antibody-drug conjugates with immunotherapy in solid tumors: Current landscape and future perspectives. Cancer Treat. Rev..

